# Identification of locally advanced rectal cancer-related genes based on transcriptome and mendelian randomization analysis with biological validation

**DOI:** 10.3389/fimmu.2025.1675788

**Published:** 2025-11-25

**Authors:** Tianhao Lao, Jie Yang, Jie Wang, Yongqiang Wang, Qianyun Ma, Hua Chen

**Affiliations:** 1 Department of General Surgery, Affiliated Kunshan Hospital of Jiangsu University, Kunshan, China; 2 School of Medicine, Jiangsu University, Zhenjiang, China; 3 Department of Emergency Surgery, Kunshan Hospital of Traditional Chinese Medicine, Kunshan Affiliated Hospital of Nanjing University of Chinese Medicine, Kunshan, China; 4 Department of Gastroenterology, The Affiliated Huaian No.1 People’s Hospital of Nanjing Medical University, Huaian, China

**Keywords:** locally advanced rectal cancer, mendelian randomization, co-expressed genes, immune cell infiltration, expression quantitative trait loci

## Abstract

**Background:**

Colorectal cancer remains one of the leading causes of cancer-related mortality worldwide, with locally advanced rectal cancer (LARC) representing a particularly challenging clinical subset. Deciphering the molecular mechanisms of LARC is essential for the development of more effective and personalized therapeutic strategies.

**Methods:**

Gene expression profiles from the Gene Expression Omnibus (GEO) database were analyzed to identify differentially expressed genes (DEGs) in LARC. Mendelian randomization (MR) analysis was subsequently performed using publicly available eQTL data to assess potential causal relationships between these DEGs and LARC. External validation of DEGs was conducted using data from the Cancer Genome Atlas (TCGA). Enrichment analyses were further conducted to explore the biological significance. To characterize the immunological landscape of LARC, immune cell infiltration and scRNA-seq analyses were employed. Survival analysis was conducted to evaluate the prognostic relevance. Finally, functional assays were performed on selected gene to validate its roles in LARC pathogenesis *in vitro*.

**Results:**

A total of 1, 113 upregulated and 1, 233 downregulated genes were identified in LARC. MR analysis, combined with external TCGA validation, revealed 9 significant co-expressed genes (CEGs) potentially involved in LARC pathogenesis. These CEGs were primarily enriched in pathways related to immune regulation, oxidative stress response, ERK1/ERK2 and JAK-STAT signaling, as well as cancer metabolism and therapeutic resistance. Immune infiltration and scRNA-seq analyses revealed notable alterations in the tumor microenvironment and distinct expression patterns of the CEGs. Notably, *in vitro* functional assays of the less-reported gene SLC19A1 demonstrated its role in promoting LARC progression.

**Conclusion:**

This study offers new insights into the molecular mechanisms underlying LARC pathogenesis and identifies potential therapeutic targets.

## 1 Introduction

Colorectal cancer (CRC) is the third most commonly diagnosed malignancy and the second leading cause of cancer-related death worldwide (1). Among these, locally advanced rectal cancer (LARC), defined as tumors that invade beyond the muscularis propria (T3–T4) or involve regional lymph nodes (N+), but without distant metastasis (M0), poses a significant clinical challenge. LARC accounts for approximately 70–80% of newly diagnosed rectal cancer cases and is associated with a high risk of both local recurrence and distant metastasis, underscoring the urgent need for improved prognostic markers and therapeutic strategies (2). Current standard treatment follows a multimodal approach consisting of neoadjuvant chemoradiotherapy (nCRT), total mesorectal excision, and adjuvant chemotherapy. nCRT consistently promotes tumor regression, increases R0 resection rates, and reduces the likelihood of locoregional recurrence (3–5). However, long-term outcomes for patients with LARC remain unsatisfactory, largely due to the high incidence of distant metastasis and the emergence of primary or acquired resistance to therapy. These persistent clinical challenges highlight the urgent need for reliable predictive biomarkers and individualized precision therapeutic approaches (6, 7). Emerging treatment modalities, including immune checkpoint inhibitors and precision-targeted therapies, have shown promising potential in improving outcomes for LARC. Nonetheless, the complex molecular mechanisms that drive tumor progression and therapeutic resistance remain poorly understood (8, 9). Therefore, a comprehensive characterization of the genomic and molecular landscape of LARC may facilitate the development of more effective and personalized treatment strategies (10).

Mendelian randomization (MR) utilizes germline genetic variants as instrumental variables (IVs) to infer causal relationships between exposures and disease outcomes, thereby minimizing the effects of reverse causation and residual confounding commonly encountered in traditional observational studies (11, 12). In this study, we integrated transcriptomic data from the Gene Expression Omnibus (GEO) to identify differentially expressed genes (DEGs) potentially involved in the pathogenesis of LARC. By combining these data with genome-wide association study (GWAS) results, we performed MR analysis to identify co-expressed genes (CEGs) that are causally associated with LARC.

Gene Ontology (GO) and Kyoto Encyclopedia of Genes and Genomes (KEGG) enrichment analyses were performed to explore the biological processes and signaling pathways associated with these genes. Furthermore, we evaluated the immunological landscape of LARC by analyzing immune cell infiltration and assessing the correlations between CEGs and specific immune cell subsets within the tumor microenvironment. In addition, single-cell RNA sequencing (scRNA-seq) data were used to validate the expression patterns of CEGs at single-cell resolution. The prognostic value of the CEGs was subsequently validated through survival analysis using an independent external cohort. Finally, *in vitro* functional validation was performed through gene knockdown and cell proliferation assays.

## 2 Materials and methods

### 2.1 Data collection

The inclusion criteria for selecting LARC microarray datasets were as follows: (1) each dataset must contain at least 60 samples, including a minimum of 30 LARC samples and 30 normal tissue samples; (2) the dataset must not include any chemically treated samples; and (3) raw data or array-based gene expression profiling data must be publicly available. Based on these criteria, two eligible LARC microarray datasets were selected for this study: GSE87211 and GSE20842. Together, these datasets comprised 268 LARC samples and 225 control samples. All gene expression matrices and corresponding platform probe annotations were obtained from the GEO database (https://www.ncbi.nlm.nih.gov/geo/). The RNA-seq data of CRC samples were obtained from The Cancer Genome Atlas (TCGA) database (https://portal.gdc.cancer.gov/) for external validation. In addition, RNA-seq data and survival data of 156 samples from the GSE103479 dataset were used for survival analysis validation.

### 2.2 Identification of DEGs

The two datasets were independently read and preprocessed using R software (version 4.3.1). Probe IDs were mapped to gene symbols based on annotation files obtained from the GEO database. For genes represented by multiple probes, only the probe with the highest average expression was retained. Log2 transformation was applied automatically based on quantile statistics. Expression values were normalized using the normalizeBetweenArrays function from the limma package (version 3.60.3). The processed datasets were then saved and merged into a single combined dataset. To account for potential batch effects between datasets, principal component analysis (PCA) was conducted before and after correction to evaluate the impact.

Differentially expressed genes (DEGs) were identified using the classical Bayesian method implemented in the limma package, with significance thresholds set at an adjusted P-value<0.05 and |LogFC|>1. Volcano and heatmaps plots of the identified DEGs were generated using the ggplot2 and pheatmap packages.

### 2.3 Exposure factors and outcome variables

In this study, expression quantitative trait loci (eQTL) data from blood were obtained from the IEU Open GWAS database (https://gwas.mrcieu.ac.uk/) and used as exposure variables (13). Single nucleotide polymorphisms (SNPs) were selected based on the following criteria: (1) a genome-wide significant association with the exposure trait (P<5e-08); (2) no linkage disequilibrium with other SNPs (r²<0.001, kb=10000); and (3) an F-statistic greater than 10, calculated using allele frequency, effect size, standard error, and sample size. SNPs meeting all three conditions were retained as instrumental variables (IVs) for Mendelian randomization (MR) analysis. GWAS data for CRC were retrieved from the GWAS Catalog (https://www.ebi.ac.uk/gwas/home; accession number GCST90013862). This dataset includes 407, 746 samples and 11, 039, 202 SNPs and was used as the outcome dataset in the MR analysis.

### 2.4 MR analysis

Mendelian randomization (MR) analysis was performed using the TwoSampleMR package (version 0.6.4) in R. The inverse variance weighted (IVW) method was employed as the primary analytical approach, while MR Egger, weighted median, simple mode, and weighted mode methods were used as complementary analyses. Genes were selected based on the following criteria: (1) P-value<0.05 in the IVW analysis; (2) consistent direction of odds ratios (ORs) across all five methods; (3) adjusted P-value<0.05 after false discovery rate (FDR) correction; and (4) no evidence of horizontal pleiotropy, as indicated by pleiotropy testing (P>0.05). Genes that met all these criteria were intersected with DEGs to identify candidate genes, which were further classified as upregulated or downregulated. To assess the robustness of the MR results, heterogeneity testing, pleiotropy analysis, and leave-one-out sensitivity analysis were conducted. The results were visualized using scatter plots, forest plots, and leave-one-out plots. Additionally, chromosomal localization of the final candidate genes was analyzed and visualized to provide genomic context.

### 2.5 External validation of TCGA database

To externally validate the expression of candidate genes identified through MR analysis and differential expression analysis, RNA-seq data from CRC samples in the TCGA database were analyzed. Differential expression between tumor and normal tissue samples was assessed using the DESeq2 package to determine whether the candidate genes exhibited significant expression changes. Genes showing statistically significant differences were designated as co-expressed genes (CEGs).

### 2.6 GO/KEGG enrichment analysis

To further investigate the potential biological functions and pathways associated with the CEGs, Gene Ontology (GO) functional annotation and Kyoto Encyclopedia of Genes and Genomes (KEGG) pathway enrichment analyses were performed using the clusterProfiler package (version 4.12.0), with statistical significance set at P<0.05.

### 2.7 Immune cell infiltration analysis

In this study, the CIBERSORT algorithm (version 1.03) was applied to evaluate the infiltration levels of 22 immune cell types in LARC tumor and normal tissue samples. Statistical comparisons between groups were performed using the Wilcoxon rank-sum test. To control for multiple testing across 22 immune cell types, p-values were adjusted using the Benjamini–Hochberg false discovery rate (FDR) method. Additionally, Spearman correlation analysis was conducted between the CEGs and immune cell proportions to explore the potential regulatory roles of CEGs within the tumor immune microenvironment.

### 2.8 Gene set enrichment analysis

Gene Set Enrichment Analysis (GSEA) was conducted to investigate functional pathway activities associated with the expression levels of individual genes. CEGs with significant roles in immune regulation were selected for detailed GSEA. All genes were ranked by log2 fold change and analyzed against curated gene sets from the MSigDB database using the clusterProfiler package. Pathways with a P-value<0.05 were considered significantly enriched.

### 2.9 single-cell RNA sequencing analysis

To evaluate the single-cell expression patterns of CEGs, single-cell RNA sequencing (scRNA-seq) data from the GSE166555 dataset were retrieved and analyzed using the Tumor Immune Single-cell Hub 2 (TISCH2) database (http://tisch.compbio.cn/home/) (14).

### 2.10 Survival analysis

To evaluate the prognostic significance of CEGs, RNA-seq data and overall survival data were obtained from the GSE103479 dataset. Kaplan–Meier survival analysis was performed using the survival and survminer packages in R, with survival differences assessed by the log-rank test. Genes with a P-value<0.05 were considered significantly associated with patient prognosis.

### 2.11 Cells and culture conditions

Human CRC cell lines (HCT116, SW480) were obtained from the American Type Culture Collection (ATCC). All cells were cultured in DMEM medium, supplemented with 10% fetal bovine serum, 1% penicillin-streptomycin at 37˚C with 5% CO_2_.

### 2.12 Cell transfection

We used the lentiviral vector pLKO.1-TRC-EGFP to deliver shRNA targeting SLC19A1, as well as a non-silencing control. The shRNA sequence targeting SLC19A1 was CGACGGTGTTCAGAATGTGAA. Cell transfection was performed using the calcium chloride method with the indicated plasmids.

### 2.13 Real-time quantitative polymerase chain reaction

RT-qPCR measured the mRNA expression of SLC19A1. Total RNA was extracted from CRC cell lines using TRIzol reagents. qPCR was performed at 95°C for 10 minutes, followed by 40 cycles of amplification at 95°C for 15 s, 60°C for the 60 s, and 95°C for 1 s. Reverse transcription was performed at 25°C for 10 minutes, 42°C for 15 minutes, and 85°C for 5 minutes. The GAPDH mRNA was used as an internal control. The sequences of the oligonucleotides used in this study were as follows: SLC19A1, forward, 5’-CTTTGCCACCATCGTCAAGACC-3’ and reverse, 5’-GGACAGGATCAGGAAGTACAC-3’; GAPDH, forward, 5’-GAGAAGGCTGGGGC- TCATTT-3’ and reverse, 5’-ATGAC-GAACATGGGGGCATC-3’.

### 2.14 Cell proliferation assay

Cell proliferation was assessed using the Cell Counting Kit-8 (CCK-8). Approximately 5×10³ cells per well were seeded into 96-well plates with flat bottoms and cultured in complete medium. At the indicated time points (0, 24, 48, and 72 hours) after transfection, 10 μL of CCK-8 solution was added to each well. The cells were then incubated at 37°C for 2–4 hours. The absorbance at 450 nm (OD_450_) was measured using a microplate reader. All experiments were performed in triplicate.

### 2.15 Colony formation assay

HCT116 and SW480 cells were trypsinized, resuspended in complete growth medium, and seeded into 6-well plates at a density of 500 cells per well. After 14 days of incubation, cells were fixed with 100% methanol for 15 minutes and stained with 0.5% crystal violet solution for 30 minutes. Plates were air-dried, and colonies containing>50 cells were manually counted. All experiments were performed in triplicate.

### 2.16 Statistical analysis

**Figure 1:**
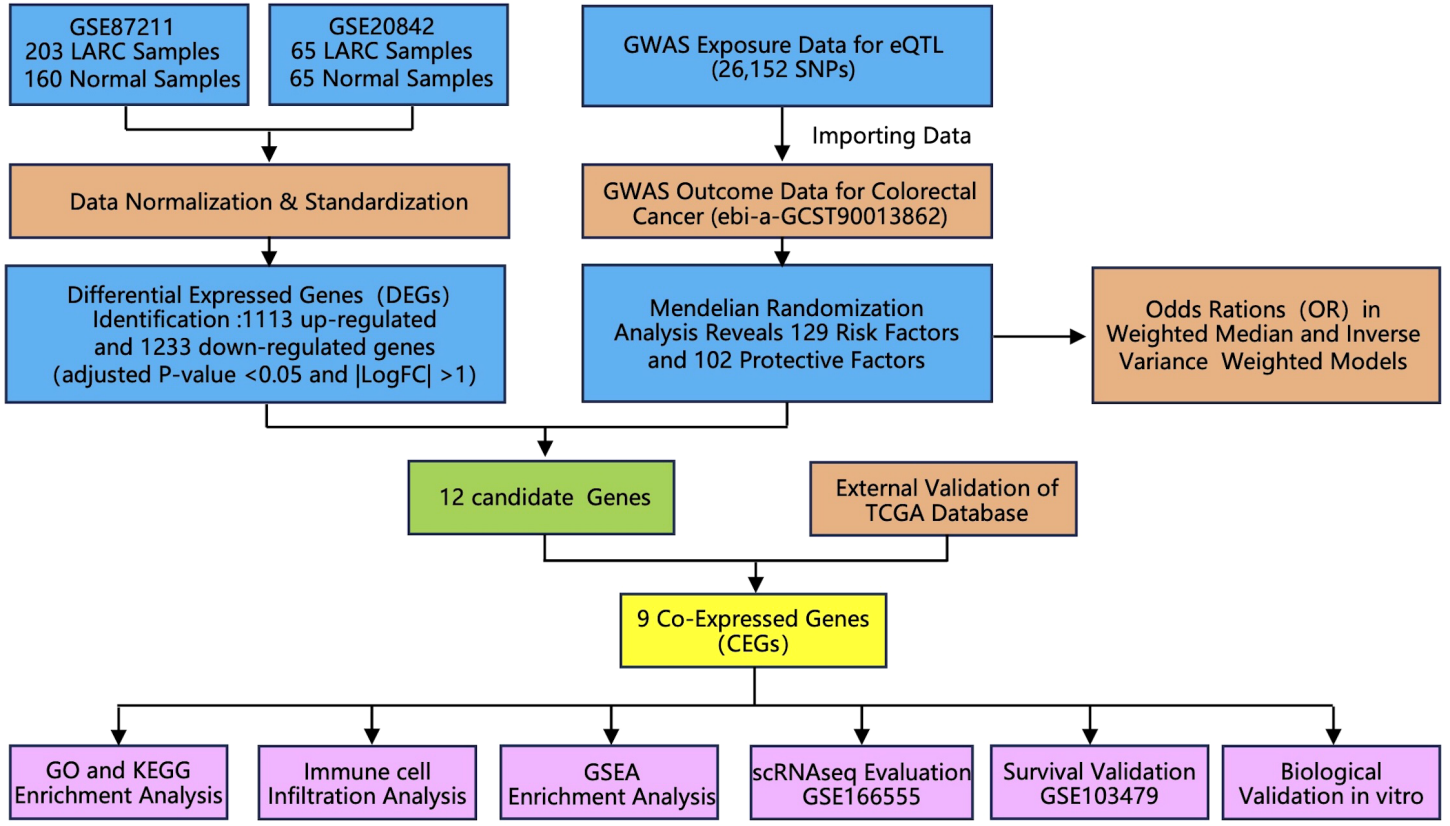
Flowchart of the study design. Abbreviations: LARC, locally advanced rectal cancer; GWAS, genome-wide association study; TCGA, The Cancer Genome Atlas; GO, gene ontology; KEGG, Kyoto encyclopedia of genes and genomes; GSEA, Gene set enrichment analysis.

In this study, the SPSS 22.0 software was used to analyze the statistical differences. We used the Student’s t-test or ANOVA to analyze group differences. All information is presented as the mean standard deviation. A P-value of less than 0.05 was regarded as statistically significant. The logical sequence of the analysis performed in the article is illustrated in **Figure 1**, as depicted in the flowchart.

## 3 Results

### 3.1 Identification of DEGs

**Figure 2:**
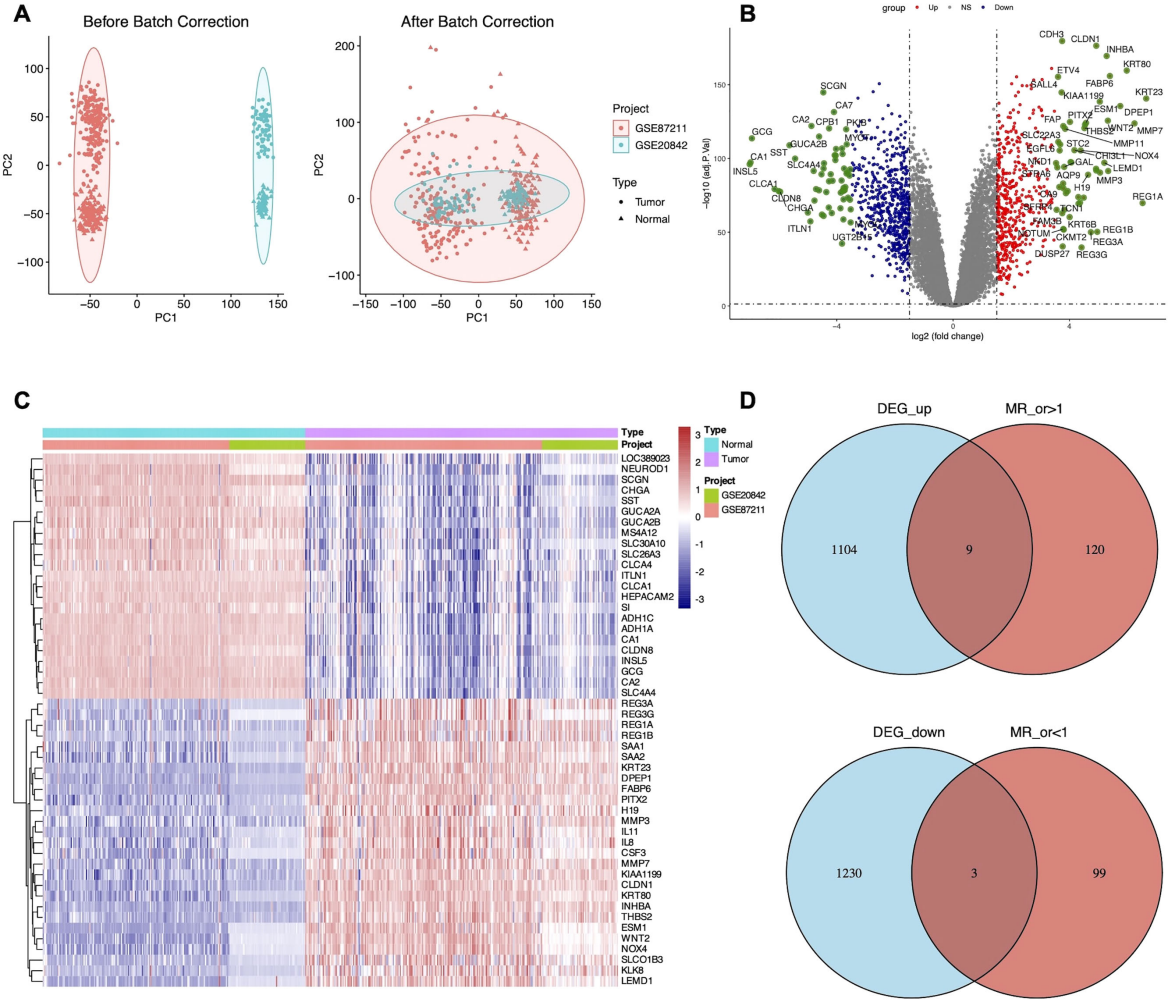
Identification of 12 candidate DEGs. **(A)** Principal component analysis (PCA) of LARC datasets before and after batch effect correction. **(B)** Volcano plot of DEGs between GSE87211 and GSE20842 datasets of LARC with the cut-off criteria of adjusted P-value <0.05 and |LogFC| >1. **(C)** Heatmap displaying the top 50 significant DEGs. **(D)** Venn diagram showing the overlap of nine up-regulated and three down-regulated candidate DEGs.

Gene expression data from two GEO datasets were normalized and quality-controlled prior to integration into a single dataset. Batch effects were corrected using principal component analysis (PCA), as illustrated in **Figure 2A**. A total of 2, 346 DEGs were identified, comprising 1, 113 upregulated and 1, 233 downregulated genes (**Supplementary Table S1**). The overall distribution of DEGs is visualized in a volcano plot (**Figure 2B**). A heatmap displaying the top 50 upregulated and downregulated DEGs, selected based on fold change and adjusted P-value, revealed distinct expression patterns between groups (**Figure 2C**).

### 3.2 MR analysis

**Figure 3:**
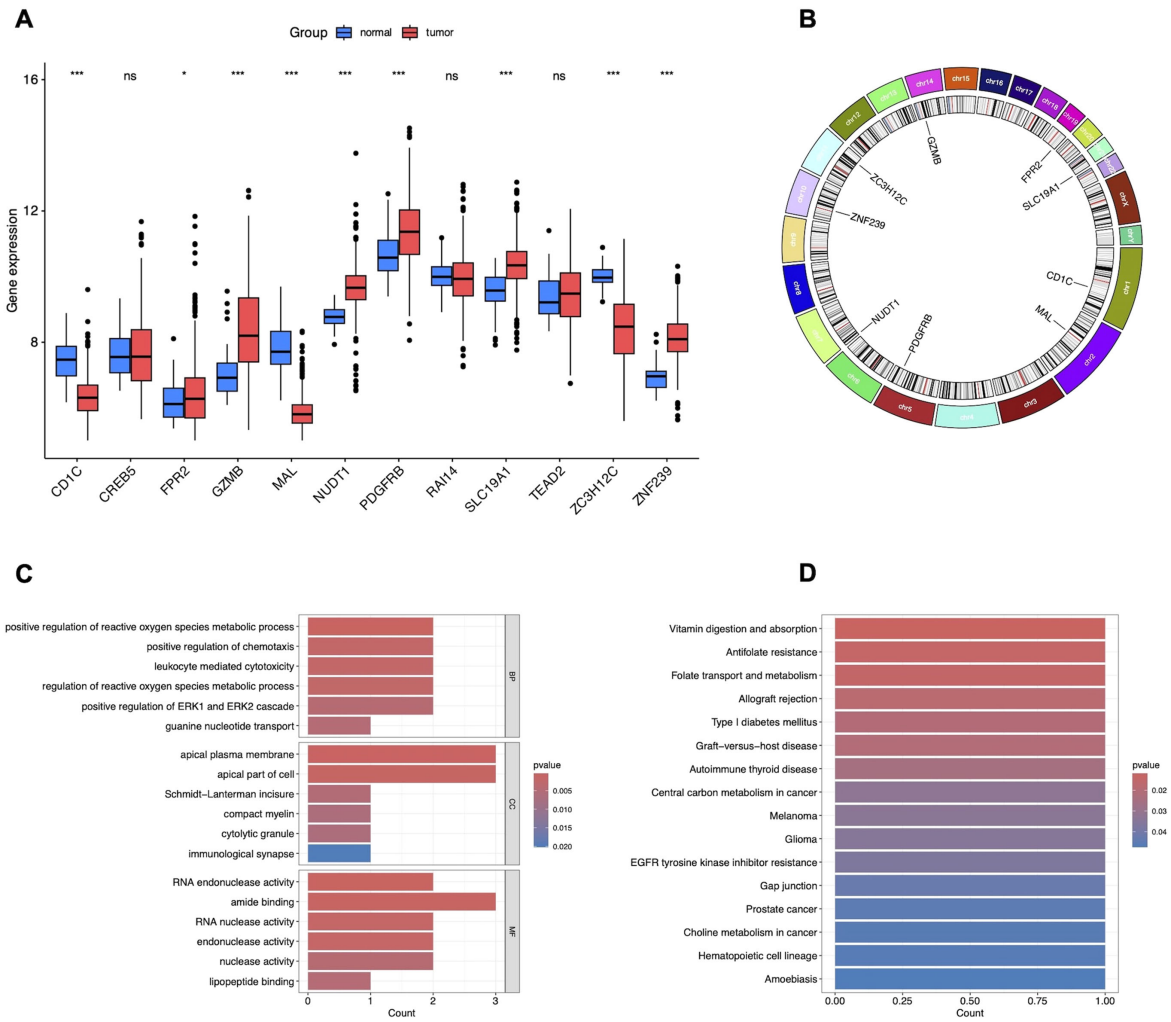
Validation and functional enrichment of nine CEGs. **(A)** Box plots showing the expression levels of 12 candidate DEGs in TCGA-CRC. *P<0.05, ***P<0.001. **(B)** Circos plot of CEGs. **(C)** GO enrichment analysis of CEGs. **(D)** KEGG pathway enrichment analysis of CEGs.

To investigate the causal relationship between genetic variants and CRC, we performed a Mendelian randomization (MR) analysis, adhering to the three core assumptions of MR: relevance, independence, and exclusion restriction. Using stringent filtering criteria based on F-statistics (F>10), we identified 26, 152 single nucleotide polymorphisms (SNPs) as robust instrumental variables (IVs), detailed in **Supplementary Table S2**. MR analyses were then conducted to assess the associations between these IVs and CRC risk. Based on the filtering criteria, we identified 102 protective factors with odds ratios (OR)<1 and 129 risk factors with OR>1 (**Supplementary Table S3**). By intersecting the MR results with 2, 346 differentially expressed genes (DEGs), we identified 12 candidate genes (**Figure 2D**). External validation using TCGA data revealed nine co-expressed genes (CEGs), including six co-expressed upregulated genes (FPR2, GZMB, NUDT1, PDGFRB, SLC19A1, and ZNF239) and three co-expressed downregulated genes (CD1C, MAL, and ZC3H12C) (**Figure 3A**).

**Figure 4:**
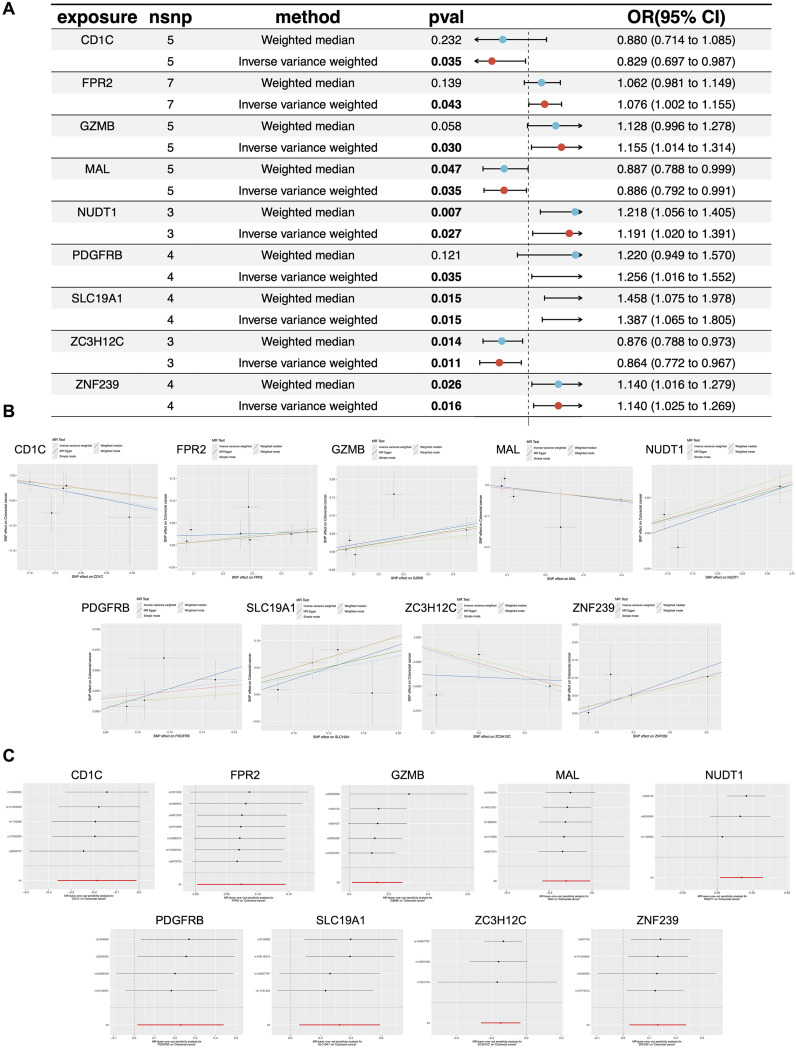
MR analysis of CEGs. **(A)** Forest plots displaying risk estimates for the nine co-expressed genes using both MR-Egger (ME) and inverse variance weighted (IVW) methods. **(B)** Scatter plot of SNP-level MR results for the nine genes in relation to CRC risk. **(C)** Leave-one-out sensitivity analysis plot, indicating influential outliers. Consistent evidence of a causal association remained after excluding each variant. Effect estimates are expressed per standard deviation increase in the exposure gene, with 95% confidence intervals shown as error bars.

We constructed forest plots to display the odds ratios (ORs) calculated by two Mendelian randomization models: Weighted Median (WM) and Inverse Variance Weighted (IVW) methods. Specifically, NUDT1 (WM: OR (95%CI)=1.218 (1.056 to 1.405), P = 0.007; IVW: OR (95%CI)=1.091 (1.020 to 1.391), P = 0.027), SLC19A1 (WM: OR (95%CI)=1.458 (1.075 to 1.978), P = 0.015; IVW: OR (95%CI)=1.387 (1.065 to 1.805), P = 0.015) and ZNF239 (WM: OR (95%CI)=1.140 (1.016 to 1.279), P = 0.026; IVW: OR (95%CI)=1.140 (1.025 to 1.269), P = 0.016) exhibited significant positive correlations with CRC risk. In contrast, MAL (WM: OR (95%CI)=0.887 (0.788 to 0.999), P = 0.047; IVW: OR (95%CI)=0.886 (0.729 to 0.991), P = 0.035) and ZC3H12C (WM: OR (95%CI)=0.876 (0.788 to 0.973), P = 0.014; IVW: OR (95%CI)=0.864 (0.772 to 0.967), P = 0.011) demonstrated significant negative correlations with CRC risk (**Figure 4A**). The scatter plot illustrates the analysis of FPR2, GZMB, NUDT1, PDGFRB, SLC19A1, and ZNF239 genes as risk factors, and CD1C, MAL, and ZC3H12C genes as protective factors, based on MR analysis for CRC risk (**Figure 4B**). Even after excluding SNPs located within these genes, consistent evidence of a causal relationship between these genes and CRC risk was observed (**Figure 4C**).

### 3.3 GO/KEGG enrichment analysis

To explore the biological functions and pathways associated with these nine CEGs, we first mapped their chromosomal locations (**Figure 3B**) and performed enrichment analyses using GO and KEGG enrichment analysis. GO enrichment analysis revealed that the genes primarily regulate key biological processes, including the positive regulation of reactive oxygen species (ROS) metabolic processes, chemotaxis, leukocyte-mediated cytotoxicity, ROS regulation, ERK1/ERK2 signaling, guanine nucleotide transport, apical plasma membrane dynamics, Schmidt-Lanterman incisure, compact myelin formation, cytolytic granules, immunological synapses, RNA endonuclease activity, amide binding, RNA nuclease activity, endonuclease activity, nuclease activity, and lipopeptide binding, as shown in **Figure 3C**. Additionally, KEGG enrichment analysis demonstrated that these genes significantly impact pathways related to vitamin digestion and absorption, antifolate resistance, folate transport and metabolism, allograft rejection, type I diabetes mellitus, graft-versus-host disease, autoimmune thyroid disease, central carbon metabolism in cancer, melanoma, glioma, EGFR tyrosine kinase inhibitor resistance, gap junctions, prostate cancer, choline metabolism in cancer, hematopoietic cell lineage, and amoebiasis, as depicted in **Figure 3D**.

### 3.4 Immune cell infiltration analysis in LARC

**Figure 5:**
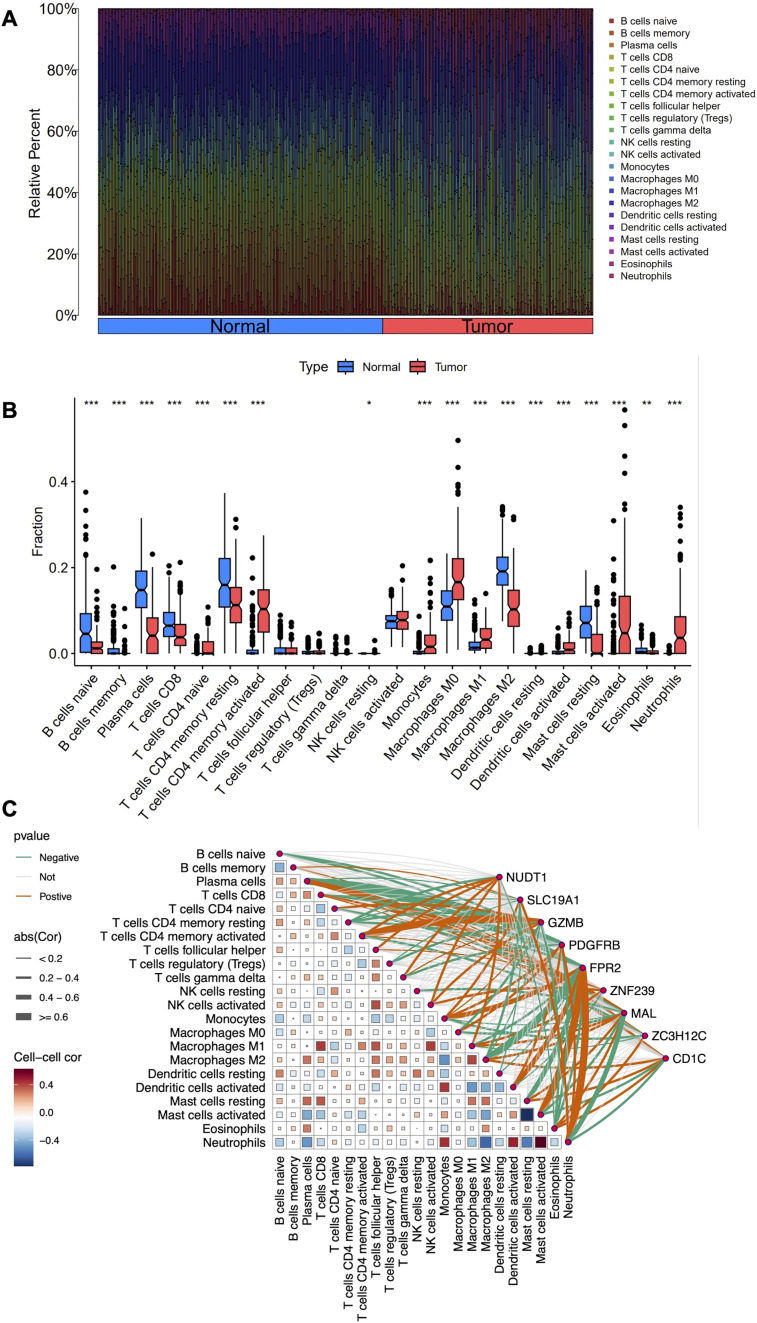
Immune cell infiltration analysis in LARC. **(A)** Stacked histogram comparing the proportions of immune cells between tumor and normal groups. **(B)** Box plots showing the differences in infiltration levels of 22 immune cell types between the two groups. **(C)** Heatmap depicting the correlation between the 22 immune cell types and the nine co-expressed genes. *P < 0.05, **P < 0.01, ***P < 0.001.

In this study, we employed the CIBERSORT algorithm to assess immune cell infiltration differences between LARC tumor and normal tissue samples, as shown in the immune cell proportion distributions (**Figure 5A**). Specifically, the proportions of B cells, plasma cells, CD8 T cells, CD4 naive and memory resting cells, M2 macrophages, and resting mast cells were significantly lower in LARC compared to normal samples. In contrast, the proportions of CD4 memory activated T cells, monocytes, M0 and M1 macrophages, activated mast cells, and neutrophils were higher in LARC samples (**Figure 5B**). Correlation analysis between co-expressed genes and immune cell infiltration revealed that FPR2 was positively correlated with neutrophils, monocytes, activated dendritic cells, and activated mast cells, while it was negatively correlated with M2 macrophages, plasma cells, and resting mast cells. PDGFRB was positively correlated with monocytes, M0 macrophages, activated dendritic cells, and neutrophils, and negatively correlated with plasma cells, naive B cells, gamma delta T cells, and eosinophils. GZMB showed a positive correlation with activated CD4 memory T cells and activated NK cells, and a negative correlation with resting CD4 memory T cells, Tregs, and M0 macrophages. CD1C was positively correlated with M2 macrophages, resting mast cells, and eosinophils, and negatively correlated with neutrophils. Finally, MAL was positively correlated with plasma cells and negatively correlated with neutrophils (**Figure 5C**).

### 3.5 GSEA of CEGs in LARC

**Figure 6:**
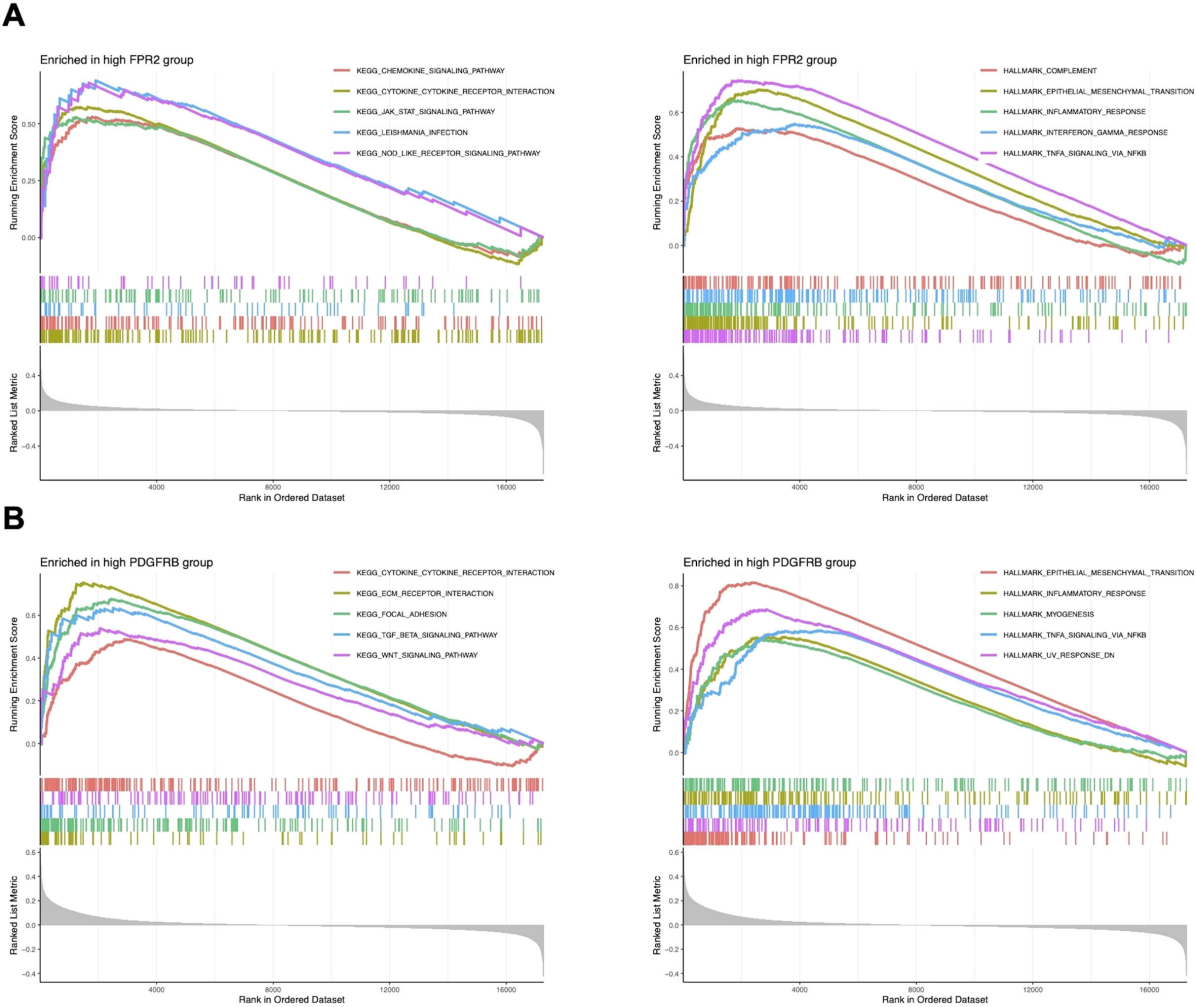
GSEA of CEGs in LARC. **(A)** The top five significantly enriched pathways and hallmark signatures in the FPR2 high-expression group. **(B)** The top five significantly enriched pathways and hallmark signatures in the PDGFRB high-expression group.

Based on the results of differential gene expression, immune cell infiltration assessment, and correlation analysis, we identified a significant association between FPR2 and PDGFRB with differential immune cell infiltration. Consequently, we further investigated the activity levels of relevant functions and pathways related to FPR2 and PDGFRB in LARC using GSEA. The GSEA results revealed that in the FPR2 high expression group, pathways associated with immune response were notably enriched. These included the chemokine signaling pathway, cytokine-cytokine receptor interaction, JAK-STAT signaling pathway, and NOD-like receptor signaling pathway, indicating FPR2’s significant role in immune modulation and inflammation within the tumor microenvironment. Additionally, pathways such as complement activation, epithelial-mesenchymal transition, inflammatory response, interferon gamma response, and TNFA signaling were also enriched, underscoring FPR2’s involvement in immune cell recruitment and inflammatory responses (**Figure 6A**). In the PDGFRB high expression group, the top five active pathways included cytokine-cytokine receptor interaction, ECM-receptor interaction, focal adhesion, and TGF-β signaling pathway, and WNT signaling pathway, all of which are critical for cell migration, adhesion, and immune system modulation in tumor progression. Furthermore, epithelial-mesenchymal transition, inflammatory response, myogenesis, and TNFA signaling were significantly enriched, suggesting that high PDGFRB expression is linked to processes involved in tumor metastasis and immune regulation (**Figure 6B**). These findings suggest that FPR2 and PDGFRB may serve as potential biomarkers or therapeutic targets for LARC, providing deeper insights into the molecular mechanisms driving the disease.

### 3.6 Single-cell expression patterns of CEGs

**Figure 7:**
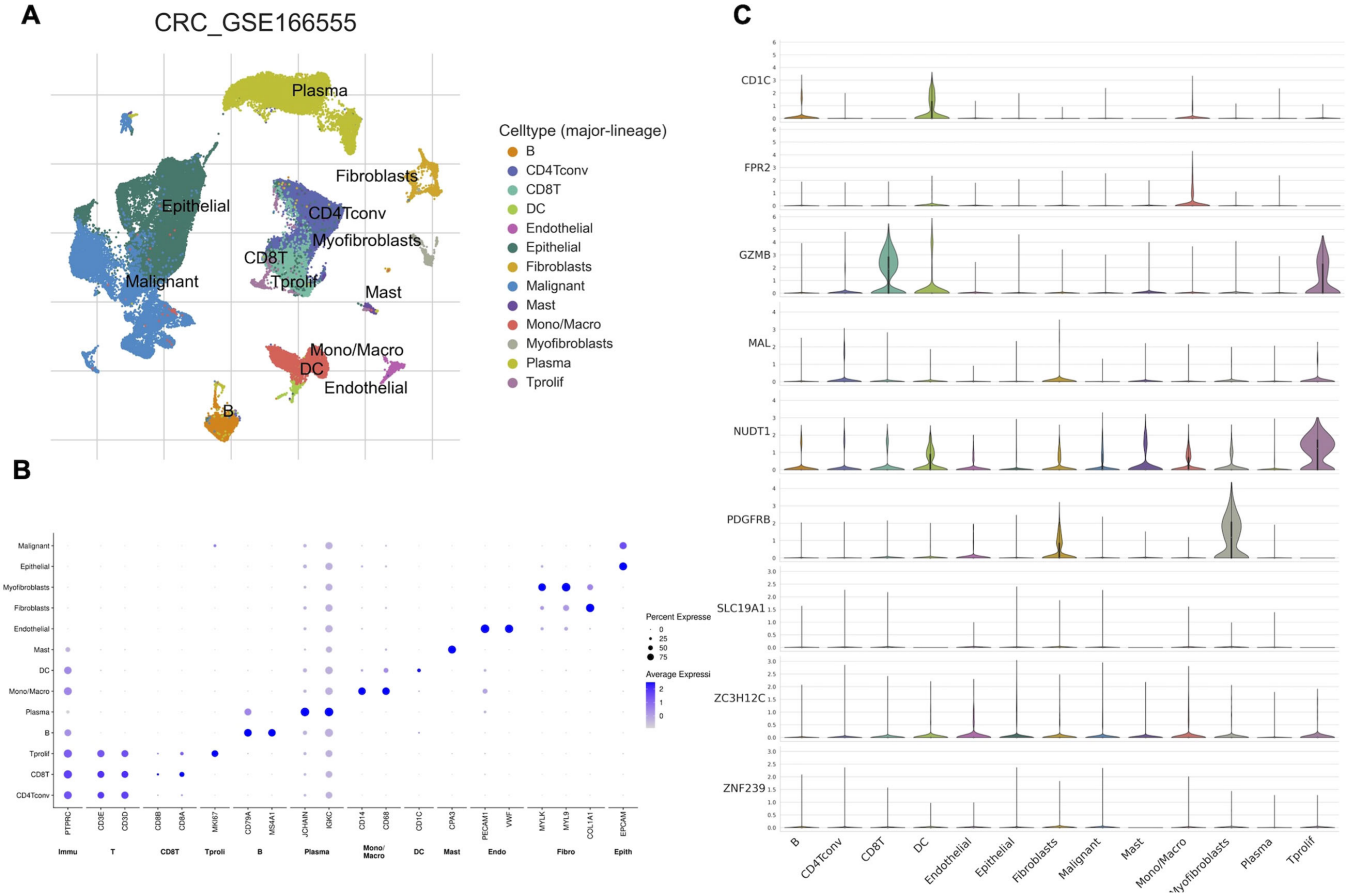
Single-cell expression patterns of CEGs. **(A)** UMAP plot illustrating cell type clustering in the GSE166555 dataset. **(B)** Dot plot showing the expression of marker genes across distinct cell types. **(C)** Violin plot showing that relative abundance of the nine co-expressed genes in each cell type.

The scRNA-seq data of CRC from the GSE166555 dataset was analyzed, revealing 13 distinct cell clusters (**Figure 7A**), which included CD4Tconv cells, CD8T cells, Tprolif cells, B cells, Plasma cells, Mono/Macro cells, dendritic cells (DC) cells, Mast cells, Endothelial cells, Fibroblasts cells, Myofibroblasts cells, Epithelial cells, and Malignant cells (**Figure 7B**). Additionally, the expression levels of the nine co-expressed genes in each cell type were visualized using a violin plot. This analysis revealed that CD1C was predominantly expressed in DC and B cells, FPR2 in mono/macrophage cells, GZMB in CD8T, Tprolif and DC cells, NUDT1 in Tprolif, DC and Mast cells, and PDGFRB in Myofibroblasts and Fibroblasts cells (**Figure 7C**).

### 3.7 Survival analysis of CEGs

**Figure 8:**
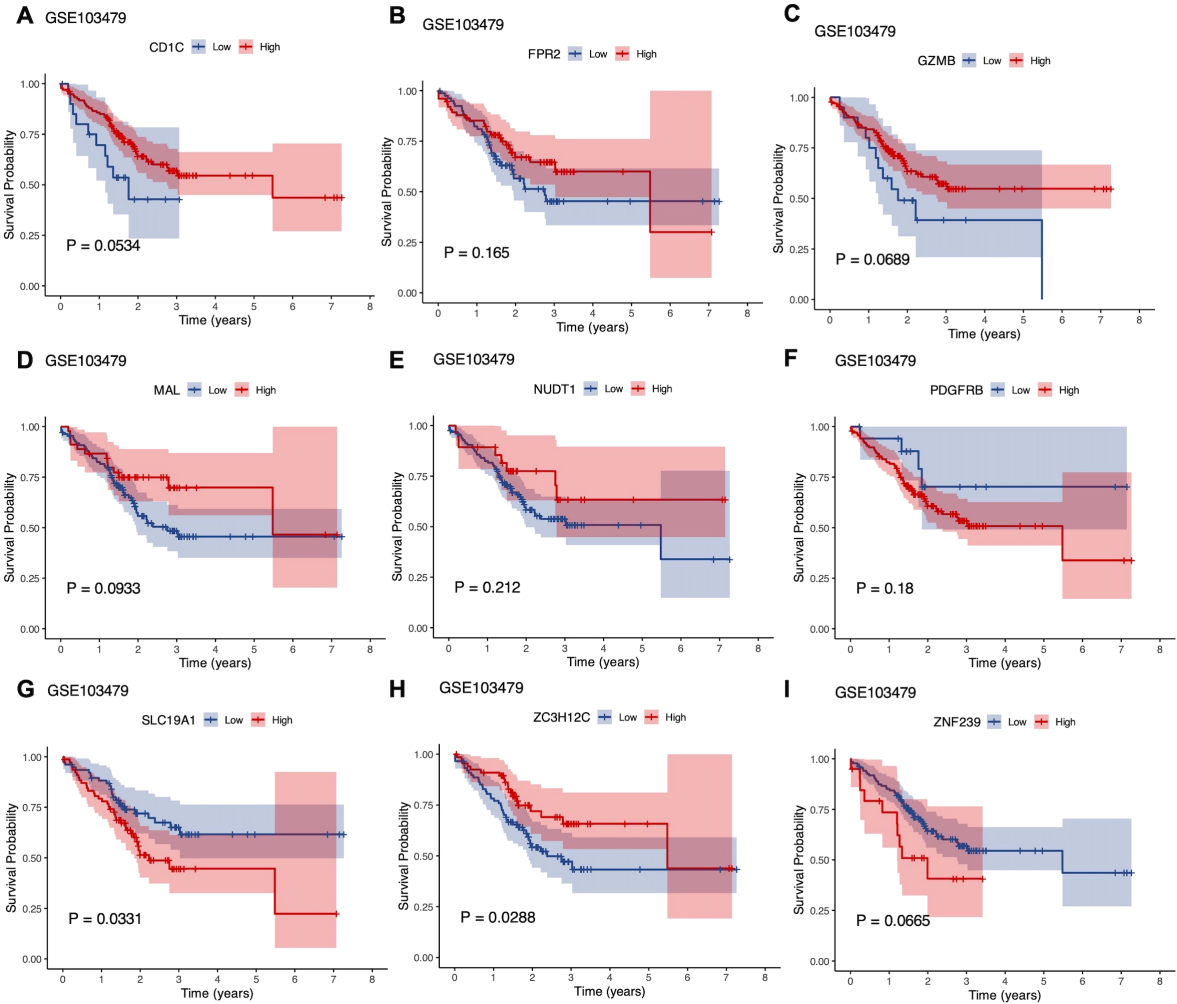
Overall survival (OS) analysis of CEGs using an external GEO dataset (GSE103479). **(A-I)** Kaplan–Meier survival curves for CD1C, FPR2, GZMB, MAL, NUDT1, PDGFRB, SLC19A1, ZC3H12C, and ZNF239 in CRC patients. Red lines indicate high-expression groups; blue lines indicate low-expression groups. The x-axis represents OS time (years), and the y-axis represents survival probability. Log-rank P-values<0.05 were considered statistically significant.

Survival analysis was conducted using the GSE103479 dataset to evaluate the prognostic relevance of the co-expressed genes. Kaplan-Meier survival curves were plotted to compare the high and low expression groups for each gene, with statistical significance assessed using the Log-rank test. Among the genes evaluated, high expression of SLC19A1 was significantly associated with poorer prognosis in CRC patients (P = 0.0331), suggesting its potential as a negative prognostic factor linked to decreased survival. Conversely, high expression of ZC3H12C was significantly correlated with better prognosis (P = 0.0288), indicating its potential as a positive prognostic factor in CRC (**Figure 8**).

### 3.8 Biological validation of SLC19A1 *in vitro*

**Figure 9:**
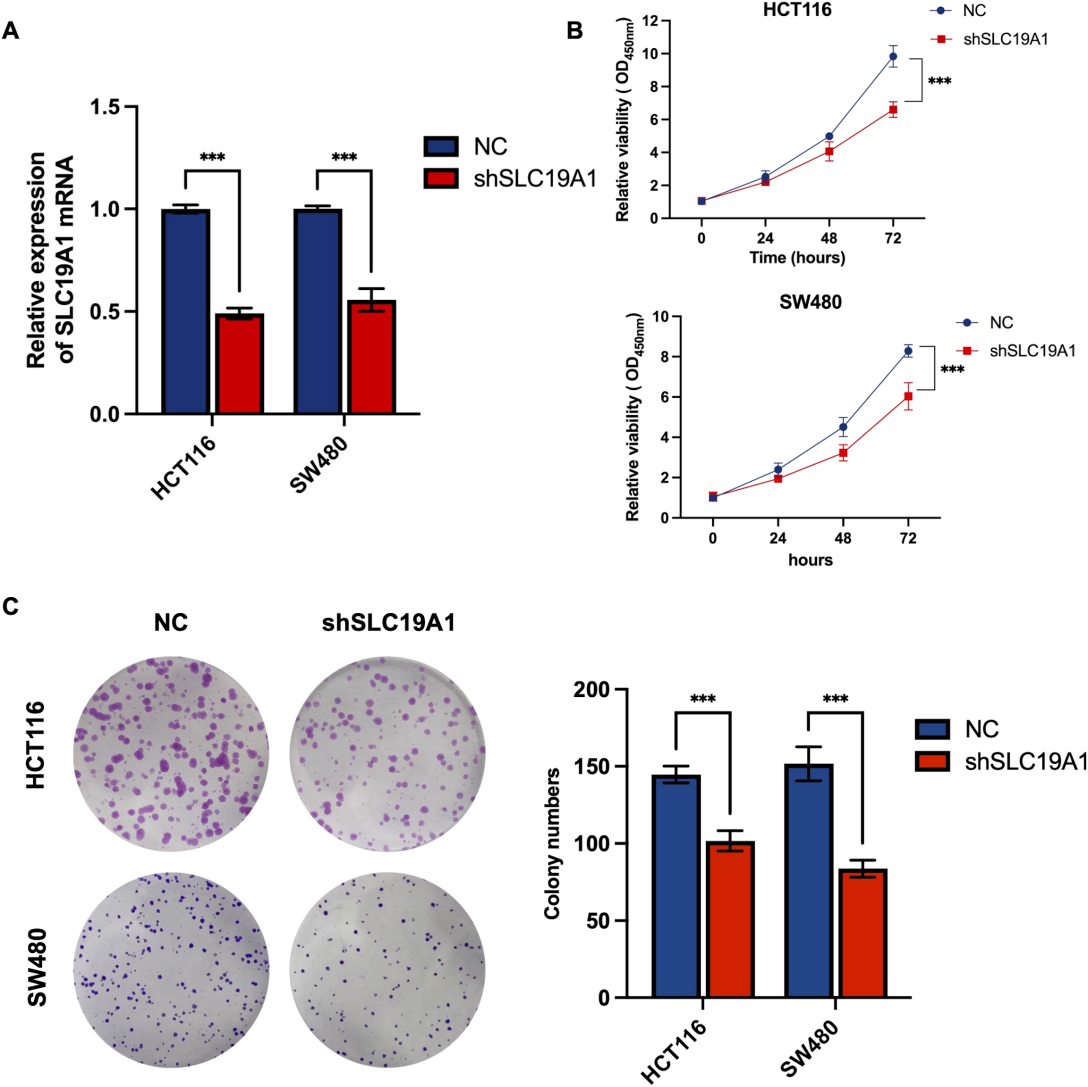
Biological validation of SLC19A1 *in vitro*. **(A)** Transfection efficiency of SLC19A1 shRNA was measured by RT-qPCR. **(B)** CCK8 assay showed the effect of SLC19A1 on the proliferation rate of HCT116 and SW480 cells. **(C)** Colony formation assay showed the effect of SLC19A1 on the colony-forming ability of HCT116 and SW480 cells. NC, negative control. ***P < 0.001.

The involvement of ZC3H12C in colorectal cancer has been previously reported (15), whereas the role of SLC19A1 remains unexplored. To investigate its potential involvement in colorectal cancer cell proliferation, we transfected HCT116 and SW480 cell lines with shRNA targeting SLC19A1. The knockdown efficiency was confirmed by RT-qPCR (**Figure 9A**). Compared with the negative control, knockdown of SLC19A1 significantly inhibited cell proliferation, as demonstrated by CCK-8 and colony formation assays (**Figures 9B, C**).

## 4 Discussion

Total neoadjuvant therapy (TNT) has been introduced to enhance treatment compliance and efficacy in patients with LARC (16, 17). However, its pathological complete response (pCR) rate remains around 40%. Recent clinical trials suggest that immunotherapy-based TNT (iTNT) achieves higher complete response rates in pMMR/MSS LARC compared to conventional chemoradiotherapy (18–20). Despite these advances, there remains an urgent need to identify novel molecular targets that can further improve and sustain therapeutic response. Our in-depth analysis has provided valuable insights into the molecular mechanisms underlying LARC. By analyzing GEO datasets, we identified 1, 113 upregulated and 1, 233 downregulated DEGs. Subsequent MR analysis, combined with external validation using TCGA data, led to the identification of key genes potentially associated with LARC. This integrative approach identified nine co-expressed genes, including six upregulated genes (FPR2, GZMB, NUDT1, PDGFRB, SLC19A1, ZNF239) and three downregulated genes (CD1C, MAL, ZC3H12C). GO and KEGG enrichment analyses revealed that the nine identified CEGs are involved in a variety of biological processes and cancer-related pathways. These include immune regulation, oxidative stress response, and signaling pathways such as ERK1/ERK2 and JAK-STAT, as well as pathways associated with cancer metabolism and therapeutic resistance. These findings suggest that the CEGs may contribute to tumor progression and immune modulation in LARC.

Immune infiltration analysis revealed distinct alterations in LARC, including decreased B cells, plasma cells, and CD8 T cells, and increased monocytes, M0/M1 macrophages, and neutrophils. Co-expressed genes such as FPR2 and PDGFRB were significantly associated with distinct immune cell subsets in the LARC microenvironment, showing positive correlations with pro-inflammatory cells such as neutrophils and monocytes. Conversely, both genes were negatively correlated with plasma cells, naïve B cells, and other immune subsets, suggesting their roles in modulating tumor-related immune responses. GSEA further revealed that FPR2 is primarily involved in inflammatory and chemokine signaling, while PDGFRB is associated with pathways regulating cell adhesion, migration, and epithelial-mesenchymal transition, underscoring their roles in immune modulation and tumor metastasis. Complementing these findings, scRNA-seq analysis showed distinct expression patterns of the co-expressed genes across specific immune and stromal cell populations, with FPR2 enriched in macrophages and PDGFRB in fibroblasts and myofibroblasts, suggesting their functional relevance within the tumor microenvironment.

FPR2 (formyl peptide receptor 2) is a G-protein-coupled receptor located on chromosome 19q13 that mediates chemotaxis and immune cell activation in response to formylated peptides (21). High expression of FPR2 has been implicated in modulating tumor-associated inflammation and promoting tumor cell migration and invasion, such as gastric and colorectal cancer (22–24). FPR2 also contributes to fibroblast homeostasis through ANXA1-mediated signaling within the tumor microenvironment (TME), and disruption of this axis facilitates the transformation of fibroblasts into cancer-associated fibroblasts (CAFs), contributing to tumor progression (25). PDGFRB (platelet-derived growth factor receptor beta) is a receptor tyrosine kinase located on chromosome 5q31.1, primarily expressed on vascular smooth muscle cells and pericytes (26). Its overexpression is linked to enhanced tumor cell proliferation, angiogenesis, and fibrosis, playing a key role in maintaining the tumor vasculature and promoting metastasis (27, 28). Furthermore, PDGFRB is involved in key signaling pathways, including the MAPK and PI3K/AKT pathways, which are crucial for cancer cell survival and migration (29).

Notably, survival analysis revealed that high expression of SLC19A1 was associated with poorer prognosis, while elevated ZC3H12C expression correlated with improved survival, underscoring their potential as prognostic biomarkers. ZC3H12C (zinc finger CCCH-type containing 12C), located on chromosome 5q33.3, is a member of the RNase family that plays a key role in modulating immune responses and cytokine production (30). It plays a critical role in maintaining immune homeostasis by regulating interferon (IFN) signaling specifically in macrophages (31). Reduced expression of ZC3H12C may disrupt the regulation of inflammatory responses and immune tolerance in the tumor microenvironment, thereby facilitating colorectal cancer progression through enhanced immune evasion and tumor cell survival (15).

Functional validation experiments demonstrated that knockdown of SLC19A1 significantly inhibited cell proliferation, suggesting that SLC19A1 may act as a potential oncogene in the pathogenesis of colorectal cancer. SLC19A1, also known as reduced folate carrier (RFC), is a crucial transporter located on chromosome 21q22 that mediates the cellular uptake of reduced folates and antifolate chemotherapeutic agents (32). Elevated SLC19A1 expression may facilitate tumor progression by promoting folate-dependent nucleotide synthesis and supporting rapid cell proliferation (33). In high-risk neuroblastoma, SLC19A1 is upregulated by MYCN, enhances methotrexate uptake, and is associated with poor prognosis, indicating its potential as a therapeutic and prognostic target (34). Previous pan-cancer profiling and multi-omics analyses have identified SLC19A1 as a novel unfavorable prognostic marker, closely associated with immune suppression and genomic instability (35). SLC19A1 has also been recognized as a biomarker associated with poor prognosis in multiple myeloma (36). Moreover, tumors with low SLC19A1 expression rely heavily on cytosolic one-carbon metabolism via SHMT1, making them potentially vulnerable to SHMT1-targeted therapies and highlighting SLC19A1 as a key metabolic marker (37). In stage III colorectal cancer, high expression of SLC19A1 and SLC46A1 correlated with improved disease-free survival in patients receiving 5-FU/leucovorin (38). Importantly, SLC19A1 functions optimally at physiological pH, whereas under acidic tumor microenvironments, folate uptake is preferentially mediated by SLC46A1 (39, 40). Additionally, SLC19A1 has also been recognized as a predictor of complete response to neoadjuvant chemotherapy in hormone-sensitive breast cancer. In particular, high SLC19A1 expression was associated with higher pCR rates in regimens including fluorouracil, anthracyclines, and taxanes (41). Extrapolating to LARC, where fluoropyrimidine-based chemoradiotherapy remains the backbone of neoadjuvant treatment, it is plausible that SLC19A1 expression may similarly stratify patients most likely to benefit. Although direct clinical validation in LARC is still needed, this highlights an important avenue for future translational research. Together, these findings indicate that while SLC19A1 may support proliferation *in vitro*, in the clinical context, its expression can enhance chemotherapy efficacy. Collectively, these findings highlight SLC19A1 as a potential prognostic biomarker and therapeutic target for improving the management of LARC.

In conclusion, our investigation has provided a valuable understanding of the genetic and immune drivers underlying LARC development, while also identifying potential novel therapeutic targets. However, these findings remain preliminary and require further validation through additional experimental studies and clinical trials to confirm the underlying mechanisms and therapeutic efficacy of these genes. Several limitations should be acknowledged. Functional validation was performed only for SLC19A1 *in vitro*, while other candidate genes were not experimentally examined, and no *in vivo* assays such as xenograft models were conducted, leaving the evidence chain incomplete. In addition, the mechanistic insights remain exploratory: although enrichment and immune infiltration analyses suggested potential pathways, further functional studies are needed to confirm these associations. Moreover, the scRNA-seq dataset GSE166555 was derived from colorectal cancer samples in general rather than specifically from LARC, which may restrict disease-specific interpretation. Future work will therefore focus on *in vivo* validation, deeper mechanistic investigations, and scRNA-seq directly from LARC tissues, as well as expanding clinical studies to confirm therapeutic efficacy. Although our current findings remain preliminary, they provide promising prospects and a strong foundation for future research to comprehensively unravel the complexity of LARC and improve patient management.

## Data Availability

The original contributions presented in the study are included in the article/**Supplementary Material**. Further inquiries can be directed to the corresponding authors.
